# Vector competence of *Aedes aegypti* from New Caledonia for the four recent circulating dengue virus serotypes

**DOI:** 10.1371/journal.pntd.0008303

**Published:** 2020-05-14

**Authors:** Olivia O’Connor, Elodie Calvez, Catherine Inizan, Nicolas Pocquet, Vincent Richard, Myrielle Dupont-Rouzeyrol

**Affiliations:** 1 Institut Pasteur de Nouvelle-Calédonie, URE Dengue et Arboviroses, Institut Pasteur International Network, Noumea, New Caledonia; 2 Institut Pasteur de Nouvelle-Calédonie, URE Entomologie Médicale, Institut Pasteur International Network, Noumea, New Caledonia; 3 Institut Pasteur de Nouvelle-Calédonie, Direction, Institut Pasteur International Network, Noumea, New Caledonia; Duke-NUS GMS, SINGAPORE

## Abstract

In New Caledonia (NC), *Aedes aegypti* is the only proven vector of dengue virus (DENV), which is the most prevalent arbovirosis in NC. Since World War II, the four DENV serotypes have circulated regularly in NC. The epidemiological profile, however, has evolved over the last ten years, with the persistence of DENV-1 circulation and the co-circulation of several DENV serotypes. The current study evaluated the ability of *Ae*. *aegypti* from NC to transmit four DENV serotypes (and two DENV-1 genotypes) isolated during recent outbreaks in NC. An *Ae*. *aegypti* F1 generation was twice independently orally challenged with each DENV strain (10^7^ FFU/ml). Infection, dissemination and transmission rates and transmission efficiency were measured at day 7 and 14 post-exposure, as well as the quantity of infectious virus particles. Mosquito infection was observed as early as 7 days post-infection. Infection rates between 18 and 58% were measured for all DENV serotypes/genotypes tested. Although dissemination rates ranged from 78 to 100%, transmission efficiencies were low, with values not exceeding 21% at 14 days post-infection for all DENV strains. This study shows that NC *Ae*. *aegypti* are moderately competent for DENV in laboratory conditions. In link with epidemiological data, these results suggest implication of other factors in the sustained circulation of DENV-1 in New Caledonia.

## Author summary

*Aedes aegypti* is the only known vector for dengue virus (DENV) in New Caledonia (NC). DENV are divided into four serotypes (DENV-1 to -4), based on their antigenic properties, these being subdivided into different genotypes. All four DENV serotypes have regularly circulated in New Caledonia in the past. An unusual persistence of DENV-1 has been observed during the last ten years, suggesting a possible preferential transmission of this DENV serotype by *Ae*. *aegypti*. In this study, we examine for the first time the ability of *Ae*. *aegypti* from New Caledonia to transmit the four circulating DENV serotypes, and more precisely two genotypes of DENV-1. Our results demonstrate that this vector is moderately competent for DENV, with slight differences observed between DENV serotype/genotype in terms of transmission. These findings suggest that other factors are at play in the sustained circulation of DENV-1 and that further vector-virus interactions studies should be undertaken to better understand the DENV epidemiological profile in NC.

## Introduction

Dengue fever is a worldwide public health concern, with approximately 390 million persons affected each year and 4 billion considered at risk [1]. A broad spectrum of clinical manifestations can be encountered, usually ranging from unapparent infections to mild-febrile illness. Severe forms of dengue, however, can occur with hemorrhagic manifestations, sometimes with a fatal outcome [2]. No specific antiviral treatment is available and dengue vaccines still need improvement [3].

Dengue virus (DENV) is a positive-sense single-stranded RNA virus belonging to the *Flaviviridae* family. Four serotypes (DENV-1 to -4) can be antigenically distinguished. Infection by one serotype does not confer prolonged immunity against the others [4]. Like many RNA viruses, dengue viruses exhibit an extensive genetic diversity which makes it possible to identify distinct genotypes within each serotype [5]. DENV are transmitted to humans by the bite of infected *Aedes* mosquitoes, such as *Aedes aegypti* which is the most common vector, or, to a lesser extent, *Aedes albopictus* [6].

New Caledonia (NC) is an island territory located in the southwest Pacific Ocean, 1,210 km east of Australia. Its climate is subtropical with two-marked seasons: hot and rainy (December to May) and cold and dry, with temperature ranging from 18°C to 35°C. As many other Pacific Island Countries and Territories (PICTs), NC has regularly experienced DENV epidemics since World War II [7, 8]. Before 2000, epidemics occurred approximately every five years and outbreaks were due to a single DENV serotype/genotype, which was replaced by another one during the next epidemic. This DENV epidemiological profile, however, has changed over the last fifteen years. Indeed, we observed an unusual persistence of DENV-1, with the co-circulation of other DENV serotypes and/or other arboviruses such as Zika virus or chikungunya virus [9]. Interestingly, in 2012, DENV-1 genotype I “Asia” was introduced in NC and replaced the previous circulating DENV-1 genotype IV “Pacific” within six months [10]. This genotypic displacement led to a major outbreak in 2013 and possibly contributed to the uninterrupted circulation of DENV-1 until 2018 [9]. Thus, all four serotypes have circulated in NC in recent years: DENV-1 between 2002 and 2018; DENV-2 in 1997–1998 and 2017–2019; DENV-3 in 2014 and 2017; and DENV-4 in 2008–2009 [9].

Although many *Aedes* species are vectors of DENV in the Pacific region [11], the only known DENV vector in NC to date is *Ae*. *aegypti*. A previous study of NC *Ae*. *Aegypti* showed that the populations of the main island are genetically homogeneous [12]. This characterization of *Ae*. *aegypti* populations is important as mosquito genetic factors may influence their competence for arboviruses [13]. Previous work demonstrated the ability of *Ae*. *aegypti* from NC to transmit chikungunya or Zika viruses [14, 15]. Few data, however, are available regarding DENV and this vector’s capacity to transmit DENV in NC has not yet been investigated except for DENV-1 genotype I “Asia” [16].

The aim of this study was to evaluate the capacity of *Ae*. *aegypti* from NC to transmit the four serotypes of DENV. One DENV strain representative of each serotype/genotype isolated in NC during the recent outbreaks was selected as follows: DENV-1 genotype I “Asia” in 2016; DENV-1 genotype IV “Pacific” in 2003; DENV-2 genotype Cosmopolitan in 1998, DENV-3 genotype I in 2014 and DENV-4 genotype IIa in 2009.

## Methods

### Ethic statements

Viruses used in this study were obtained from sera from anonymized patients who were not opposed to the secondary use of their biological sample. According to New Caledonia regulations on animal care, all the experiments performed on animals were carried out following deliberation n°79 of 29/01/1989 relating to the practice of veterinary surgery.

### Virus stock production

As part of the molecular characterization of DENV strains circulating in the south Pacific region, the envelope gene sequences of DENV strains from 1997 to 2016 were obtained as described elsewhere [10, 17, 18]. One epidemic viral strain of each DENV serotype/genotype, selected from 1997 to 2016 during NC outbreaks, was isolated from human serum after no more than three passages in C6/36 cells line (Table 1). Briefly, 50 μl of serum (or viral suspension) diluted in Leibovitz medium (Sigma-Aldrich), supplemented with 2% of fetal bovine serum (FBS, Gibco, ThermoFisher Scientific) and 10% of tryptose phosphate broth (Gibco, ThermoFisher Scientific) were inoculated onto a monolayer C6/36 cells. After 2 hours, the inoculum was removed and fresh media was added to the flask. The flask was then incubated for 5 days at 28°C.

**Table 1:** Dengue virus strains used in this study.

DENV	Year of collection	Serotype	Genotype	GenBank accession number
DENV-1 genotype I "Asia"	2016	DENV-1	I "Asia"	MN539019
DENV-1 genotype IV "Pacific"	2003	DENV-1	IV "Pacific"	MN539020
DENV-2	1998	DENV-2	Cosmopolitan	MN539021
DENV-3	2014	DENV-3	I	MN539022
DENV-4	2009	DENV-4	IIa	MN539023

### Virus titration

Viral titer of each virus was determined by immuno-fluorescent focus assay. This was repeated independently three times. A ten-fold dilution of the viral suspension was inoculated onto C6/36 cells in a 96-well plate. After one hour of incubation at 28°C, an overlay consisting of 1:1 Lebovitz medium supplemented with 10% of FBS and 3.2% of carboxyl methylcellulose (Sigma-Aldrich) was added. The plate was then incubated for 5 days at 28°C. Cells were fixed with 4% formaldehyde (Pierce, ThermoFisher Scientific) at room temperature (RT) for 20 min. After a first incubation with phosphate-buffered saline (PBS, Pan Biotech, Dutscher) 0.5% Triton X-100 (Biorad) for 10 minutes at RT, cells were washed three times with PBS and then incubated with flavivirus-specific monoclonal antibody 4G2 (hybridoma D1-4G2-4-15) for one hour at 37°C. After washing, cells were incubated for one hour at 37°C with Alexa Fluor 488 goat anti-mouse IgG (Invitrogen, ThermoFisher Scientific) at a 1:1000 dilution in PBS supplemented with 5% FBS. Foci were counted under a fluorescent microscope (Leica) and the quantity of virus obtained was expressed as Focus Forming Unit per milliliter (FFU/mL).

### Mosquito collection

Immature *Ae*. *aegypti* (larvae and pupae) were collected in Noumea in November 2016. Larvae and pupae were reared to adult forms which were maintained at 28 ± 1°C, 80 ± 10% relative humidity and 12:12h light:dark cycle, with access to 10% sucrose solution *ad libitum*. F1 generation was produced by sib-mating and collective oviposition following blood feeding.

### Oral challenge

The same population of mosquitoes was experimentally infected twice to evaluate the reproducibility of the results. Five- to seven day-old nulliparous *Ae*. *aegypti* F1 females were challenged with infectious blood-meals containing each of the different DENV serotype/genotype. Briefly, mosquitoes were allowed to feed for 20 min through a pig intestine membrane covering the base of a Hemotek system feeder containing the blood-virus mixture maintained at 37°C. The infectious blood meal consisted of a mix (2:1) of washed rabbit erythrocytes and viral suspension at 3x10^7^ FFU/mL (final concentration at 10^7^ FFU/mL). An adenosine triphosphate phagostimulant (Sigma-Aldrich), was added at a final concentration of 5mM. Fully engorged females were transferred into cardboard containers and maintained with 10% sucrose solution at 28 ± 1°C, 80% relative humidity and 12:12h light:dark cycle.

### Vector competence studies

At 7 and 14 days post-infection (dpi), a maximum of 30 *Ae*. *aegypti* females were randomly collected and chilled. The wings and legs of each were removed and the proboscis was inserted into a 20 μL filter tip filled with 5 μL of FBS. After 30 min, medium containing the saliva was added to 45 μL of Leibovitz medium and stored at -80°C. Whole mosquito carcasses were also stored at -80°C before processing. After removing the head, the body and the head of each mosquito was mechanically ground separately for 30 seconds at 6,000 rpm with metal beads in 350 μL of Leibovitz medium supplemented with 2% FBS, 10% tryptose phosphate broth and antibiotics/antifungals (100 units/mL of penicillin, 0.1 mg/mL of streptomycin and 0.25 μg/mL amphotericin B, Gibco, ThermoFisher Scientific). The resulting homogenates were then centrifuged at 10,000 *g* for 5 min. The detection of viral particles in each homogenates or saliva sample was performed by virus titration as described above. The infection rate corresponds to the proportion of mosquitoes with infected body among all tested mosquitoes. The dissemination rate is the proportion of mosquitoes with infectious head among infected mosquitoes. The transmission rate is the proportion of mosquitoes with infectious viral particles in saliva among mosquitoes with infectious head, whereas transmission efficiency is the proportion of mosquitoes with virus in saliva among all tested mosquitoes.

### Statistical analysis

Two replicate experiments were performed with the same population of mosquitoes and the same DENV strains. All percentages used for the comparison of the two experiments and the determination of the different rates and efficiency were statistically compared by Fisher’s exact test. Quantitative data corresponding to virus titers obtained from positive mosquitoes were statistically compared by two-sample Wilcoxon test. To narrow down the risk estimations of the simple dichotomous-outcome analysis, multivariate analysis for the different rates and efficiency were performed using logistic regression modeling (log-linear model): the dependent variable was the outcome expressed in two categories for each mosquito (presence or absence of virus), the independent variables were “days post-infection” (with 7 dpi as reference) and the DENV strains. All tests were performed using R software, considering *p-*values < 0.05 as significant [19].

## Results

### Reproducibility of the experiments

Two separate experiments were carried out under the same experimental conditions. In both cases, all DENV strains were able to infect mosquitoes, disseminate and be transmitted. No significant difference was found in rates obtained from these two independent vector competence experiments (Fisher’s exact test: *p*-values > 0.05) (Table 2). Consequently, both datasets were pooled for further global analysis as described hereafter.

**Table 2:** Infection, dissemination and transmission rates obtained for the two independent experiments (a and b) at 7 and 14 days post-infection (dpi) according to the four dengue serotypes.

**DENV strains**	Experiment	Days post-infection (dpi)	Number of individuals	% of infection	p-value * for infection	% of dissemination	p-value* for dissemination	% of transmission	p-value* for transmission
DENV-1 genotype I "Asia"	a	7	15	33 (5/15)	0.26	100 (5/5)	0.14	20 (1/5)	0.99
DENV-1 genotype I "Asia"	b	7	30	17 (5/30)		80 (4/5)		25 (1/4)	
DENV-1 genotype IV "Pacific"	a	7	18	39 (7/18)	0.99	29 (2/7)	0.70	0 (0/2)	0.99
DENV-1 genotype IV "Pacific"	b	7	30	40 (12/30)		42 (5/12)		20 (1/5)	
DENV-2	a	7	12	42 (5/12)	0.27	60 (3/5)	0.39	0 (0/3)	NA
DENV-2	b	7	30	23 (7/30)		57 (4/7)		0 (0/4)	
DENV-3	a	7	8	13 (1/8)	0.99	0 (0/8)	0.99	0 (0/0)	NA
DENV-3	b	7	30	20 (6/30)		50 (3/6)		0 (0/3)	
DENV-4	a	7	15	47 (7/15)	0.17	43 (3/7)	0.38	33 (1/3)	0.99
DENV-4	b	7	30	23 (7/30)		43 (3/7)		33 (1/3)	
DENV-1 genotype I "Asia"	a	14	18	33 (6/18)	0.55	100 (6/6)	0.55	67 (4/6)	0.99
DENV-1 genotype I "Asia"	b	14	30	47 (14/30)		100 (14/14)		43 (6/14)	
DENV-1 genotype IV "Pacific"	a	14	18	67 (12/18)	0.55	100 (12/12)	0.24	33 (4/12)	0.18
DENV-1 genotype IV "Pacific"	b	14	30	53 (16/30)		88 (14/16)		14 (2/14)	
DENV-2	a	14	21	52 (11/21)	0.39	100 (11/11)	0.15	36 (4/11)	0.70
DENV-2	b	14	30	37 (11/30)		82 (9/11)		44 (4/9)	
DENV-3	a	14	16	63 (10/16)	0.76	80 (8/10)	0.99	25 (2/8)	0.28
DENV-3	b	14	29	55 (16/29)		88 (14/16)		7 (1/14)	
DENV-4	a	14	15	27 (4/15)	0.45	50 (2/4)	0.99	100 (2/2)	0.25
DENV-4	b	14	30	17 (5/30)		100 (5/5)		20 (1/5)	

NA: Not Achievable / *: Value obtained when data of the two experiments were statistically compared

Infection (number of infected bodies / number of mosquitoes tested)

Dissemination (number of infected heads / number of infected bodies)

Transmission (number of infected saliva / number of infected heads)

### Infection rate

**Fig 1:**
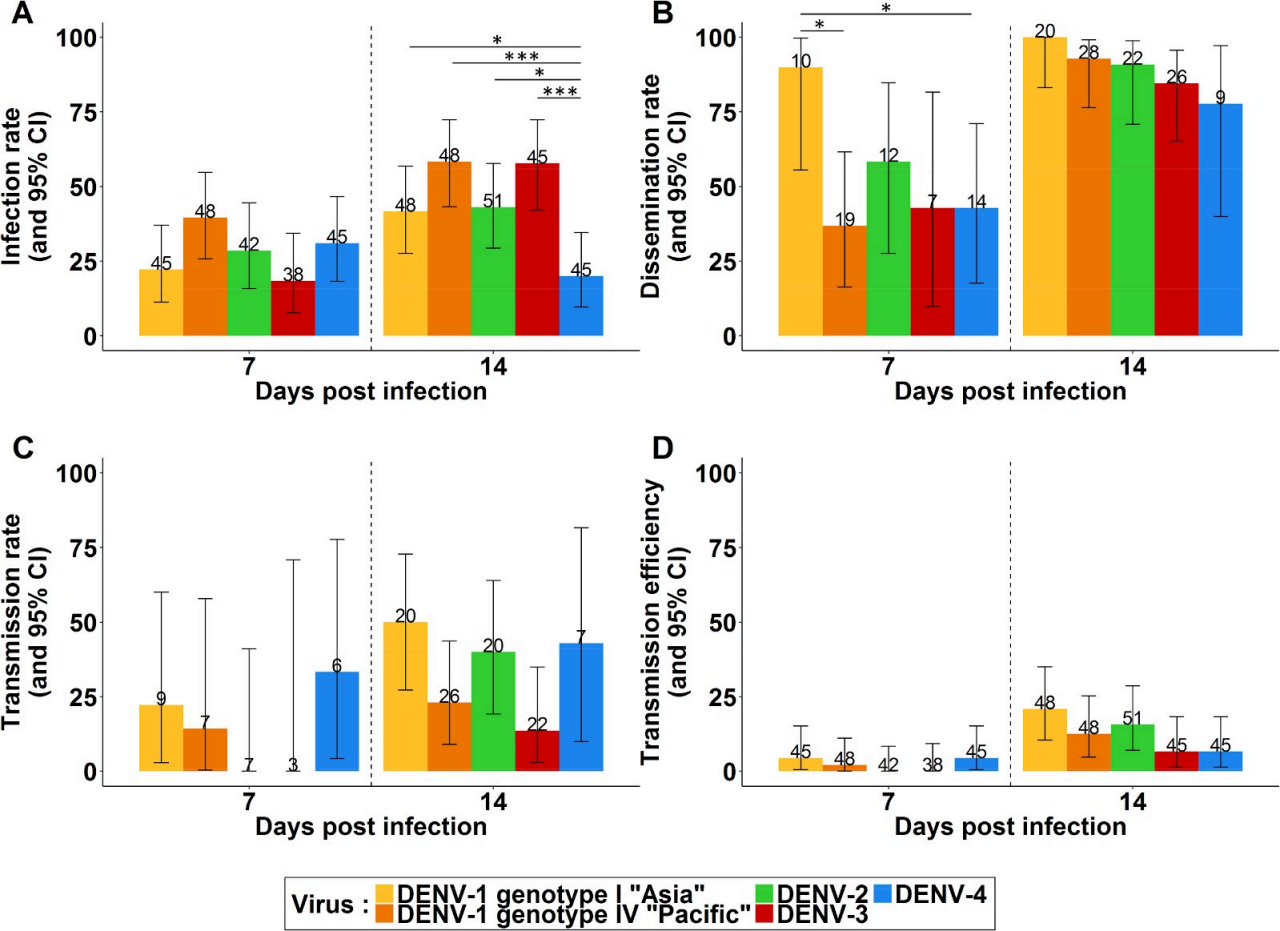
Vector competence results for *Aedes aegypti* from New Caledonia orally infected with different DENV serotype/genotype. (A) Infection rate, (B) dissemination rate, (C) transmission rate, and (D) transmission efficiency at 7 and 14 days post-infection. Error bars represent 95% confidence intervals. The number of mosquitoes tested is indicated in brackets above each bar plot. Significant differences are indicated by asterisks (Fisher’s Exact test; *p< 0.05; ** *p*< 0.01; ***p< 0.001).

Infection rates at 7 dpi ranged from 18% to 40% for the four DENV serotypes, with no significant difference (Fig 1A; S1 Table). At 14 dpi, infections rates ranged from 20% to 58%, with a significant lower infection rate for the DENV-4 strain compared to the others DENV (Fisher’s exact test: *p*-values = 0.03, < 0.001, = 0.02 and < 0.001, respectively for DENV-1 genotype I “Asia”, DENV-1 genotype IV “Pacific”, DENV-2 and DENV-3; Fig 1A). Results obtained from the log-linear model (S2 Table) showed that the infection rate was significantly higher at 14 dpi (OR = 2.04, 95%CI: 1.37–3.04). Infection rate was also significantly higher for DENV-1 genotype IV “Pacific” compared to DENV-4 as reference, after considering results of the univariate analysis (OR = 2.88, 95%CI: 1.53–5.4).

### Dissemination rate

At 7 dpi, dissemination rates ranged from 43% (DENV-3 and DENV-4) to 90% (DENV-1 genotype I “Asia”; Fig 1B). The dissemination rate for DENV-1 genotype I “Asia” was significantly higher compared to DENV-1 genotype IV “Pacific” and DENV-4 (Fisher’s exact test: *p*-values = 0.01 and 0.03 respectively). At 14 dpi, the dissemination rates ranged from 78% to 100% (Fig 1B), with no significant differences between viruses. Results obtained from the log-linear model (S2 Table) showed that dissemination rate was significantly higher at 14 dpi (OR = 9.98, 95%CI: 4.09–24.34). Dissemination rate was also significantly higher for DENV-1 genotype I “Asia” compared to DENV-4 as reference, considering results of the univariate analysis (OR = 18.45, 95%CI: 1.97–172.81).

### Transmission rate

Transmission rates at 7 dpi ranged from 0% (DENV-2 and DENV-3) to 33%, with no significant differences shown between the viruses (Fig 1C). At 14 dpi, transmission rates ranged from 14% to 50%, with a significant lower transmission rate for DENV-3 compared to DENV-1 genotype I “Asia” (Fisher’s exact test: *p*-values = 0.02; Fig 1C). Results obtained from the log-linear model (S2 Table) showed that the transmission rate was statistically higher at 14 dpi (OR = 3.57, 95%CI: 1.18–10.82). Transmission rates were also significantly higher for DENV-1 genotype I “Asia” and DENV-4 compared to DENV-3 as reference, considering results of the univariate analysis (OR = 6.67, 95%CI: 1.57–28.42 and OR = 7.1, 95%CI: 1.27–39.79 respectively).

### Transmission efficiency

No significant difference was found between DENV transmission efficiencies (Fisher’s exact test: *p*-values > 0.05; Fig 1D). The efficiency of transmission for all the DENV strains at 7 dpi did not exceed 4%, with no viral transmission observed for DENV-2 and DENV-3 infected mosquitoes. At 14 dpi, transmission efficiencies were observed for all four DENV serotypes, ranging from 7% to 21%, with the higher transmission efficiency observed for DENV-1 genotype I “Asia” infected mosquitoes. However, no significant difference was found (Fisher’s exact test: *p*-values > 0.05). Results obtained from the log-linear model (S2 Table) showed that the transmission efficiency was statistically higher at 14 dpi (OR = 6.34, 95%CI: 2.40–16.7). Transmission efficiency was also significantly higher for DENV-1 genotype I “Asia” compared to DENV-3 as reference, considering results of the univariate analysis (OR = 4.33, 95%CI: 1.15–16.2).

### Quantity of infectious viral particles among positive mosquitoes at 14 dpi

**Fig 2:**
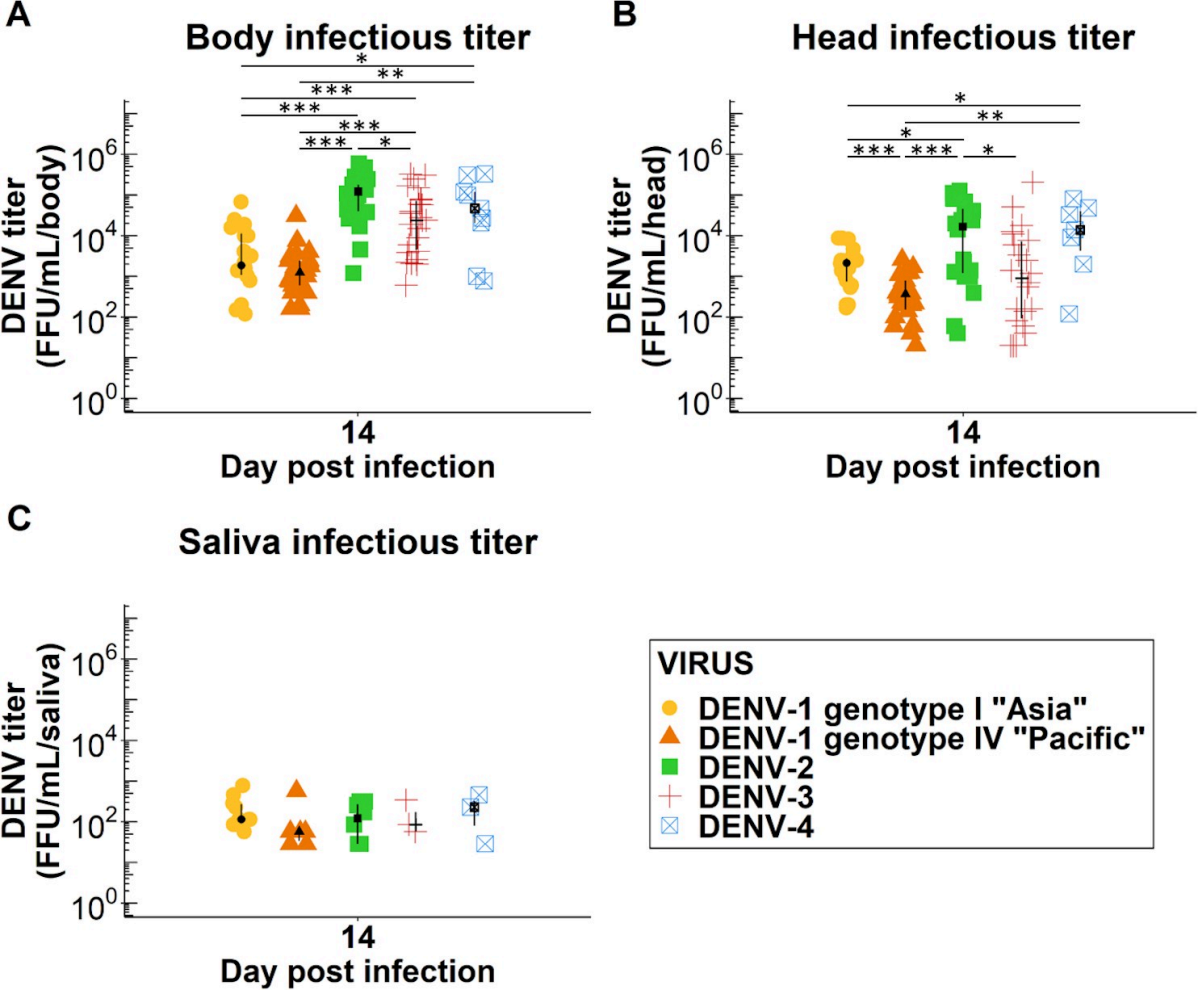
Viral titers obtained from positive bodies, heads and saliva according to the DENV serotype/genotype strain. (A) Body infection titer, (B) head infection titer and (C) saliva infection titer at 14 days post-infection. Median and interquartile range are shown for each viral strain. Significant differences are indicated by asterisks (Two-sample Wilcoxon test; *p< 0.05; ** *p*< 0.01; ***p< 0.001).

The quantity of infectious viral particles was determined for each positive mosquito at 14 dpi in parallel with the different vector competence indices (Fig 2; S3 Table). Regarding infection status, the medians of viral titer per positive bodies at 14 dpi ranged from 1.25x10^3^ to 1.22x10^5^ FFU/mL/body (Fig 2A). Mosquitoes infected with DENV-1 had a significantly lower quantity of infectious viral particles in their bodies compared to the other DENV (two-sample Wilcoxon test: *p*-value < 0.001, < 0.001, = 0.01 and < 0.001, < 0.001, = 0.002 respectively for DENV-1 genotype I “Asia” and DENV-1 genotype IV “Pacific” compared to DENV-2, DENV-3 and DENV-4). The median viral titers obtained for DENV-2 infected mosquitoes was significantly higher than that obtained from DENV-3 infected mosquitoes (two-sample Wilcoxon test: *p*-value = 0.01).

Regarding dissemination status, the medians of viral titers per head ranged from 3.7x10^2^ to 1.70x10^4^ FFU/mL/head (Fig 2B). The quantity of infectious viral particles in the heads was significantly higher for DENV-2 compared to DENV-1 genotype I “Asia”, DENV-1 genotype IV “Pacific” and DENV-3 (two-sample Wilcoxon test: *p*-value = 0.04, < 0.001 and = 0.02 respectively). DENV-1 genotype IV “Pacific” infected mosquitoes had a significantly lower viral titer in their heads compared to DENV-1 genotype I “Asia” and DENV-4 (two-sample Wilcoxon test: *p*-value < 0.001 and = 0.002 respectively). The quantity of DENV-4 infectious viral particles per heads was significantly higher than that obtained for DENV-1 genotype I “Asia” (two-sample Wilcoxon test: *p*-value = 0.03).

Regarding the transmission—the key aspect of the virus cycle in the vector—the viral titers medians ranged from 57 to 229 FFU/mL/saliva at 14 dpi (Fig 2C). No statistical analyses were performed due to the small number of saliva analyzed.

## Discussion

Since 2008, DENV circulation has increased in NC and caused recurrent outbreaks with cases detected every year. DENV-1 has been the major circulating serotype in NC until 2017, co-circulating with DENV-4 in 2009, DENV-3 in 2014, DENV-2 and -3 in 2017 and DENV-2 in 2018 [8–10]. This epidemiological profile with the prolonged circulation of DENV-1 during 15 years was particularly unusual for NC [9]. In this context, our study aimed to evaluate the ability of *Ae*. *aegypti* population from NC to transmit the four circulating DENV serotypes and two genotypes of DENV-1. Vector competence is defined as the ability of a mosquito to be infected, to disseminate and finally to be able to transmit a given virus [20]. It is based on a close interaction between the virus and the vector, which can lead to different outcomes depending on the fitness of both at the time of the experiment. To remove this bias, we performed two replicates experiments whose results were not significantly different. This observation, combined with the fact that the results obtained for DENV-1 genotype I “Asia” infected mosquitoes are equivalent to those obtained in our previous study [16], gives reliability to our results. NC *Ae*. *aegypti* is a competent vector for dengue viruses, with the detection of infectious virus in saliva from 7 dpi for three out of five DENV strains.

All four DENV serotypes can infect NC *Ae*. *aegypti* from 7 dpi. Infection rates of mosquitoes do not differ by DENV strain, except for DENV-4 which has a lower infection rate. Overall, infection rates were moderate (50–60%), suggesting the possible existence of a midgut infection barrier that impedes viral infection of mesenteric epithelial cells [21]. The dissemination rates, however, were around 50% at 7 dpi and 80–100% at 14 dpi, suggesting a weak midgut escape barrier, if any. Despite this dissemination, the transmission rates did not exceed 50% whichever the dpi, suggesting the presence of a salivary glands barrier. Our experimental design does not allow us to specify whether this barrier occurs at the entry in and/or escape from the salivary glands. Overall, the transmission efficiencies were low with no differences observed between DENV strains in pairwise comparison. These results, however, need to be modulated in view of the results obtained with the log-linear model which revealed that DENV-1 genotype I “Asia” would have a slightly higher transmission risk compared to DENV-3. In addition to these observations, infectious viral titers obtained from the infection and dissemination indices at 14 dpi showed differences between the DENV serotypes, with a tendency towards lower infectious viral titers for DENV-1. These differences do not seem to have an impact on transmission efficiencies: although the low number of positive saliva samples did not allow us to verify this fact statistically, viral titers in saliva seemed similar between all the DENV strains tested. We can suppose that this may be the result of a discontinuous release of virions in the saliva as it was shown that salivary glands are a site for replication and accumulation of DENV [22].

Vector competence studies have been performed in other countries [23] with varying results due in part to the non standardization of protocols, making their comparison difficult. Field *Ae*. *aegypti* populations were mainly used in these studies, but the DENV strains used were mostly old strains, with an overrepresentation of DENV-2 strains [23–25]. Nevertheless, the infection rates of NC *Ae*. *aegypti* we report here are lower compared to those reported in studies in Africa [24, 26], in South East Asia [27] or in the Caribbean [28], suggesting that NC *Ae*. *aegypti* are less susceptible to DENV infection. Many intrinsic and extrinsic factors [13] are involved in the modulation of *Ae*. *aegypti* vector competence, as early at the midgut infection phase. Indeed, genetic variants from both *Ae*. *aegypti* population and the virus can play a role in vector competence, whereby the outcome of the infection depends on the specific combination of mosquito and virus genotypes [29, 30]. The susceptibility of mosquitoes to infection can also be influenced by the insect microbiome [31, 32] and the microbiota interplays in the activation of mosquito immune responses. Although we evidenced moderate infection rates, the dissemination and the transmission rates are similar to those found in the different studies. The extrinsic incubation period for *Ae*. *aegypti* from NC, however, seems to be shorter, with transmission observed from 7 dpi for DENV-1 and DENV-4.

Vector competence experiments in the laboratory measure the ability of mosquitoes to transmit arboviruses. However, the experimental conditions applied in this type of experiment are only an approximation of what happens in nature. In our experimental conditions, a slightly difference was observed in terms of transmission efficiency between DENV-1 genotype I “Asia” and DENV-3. Several factors, however, need to be considered. Indeed, only a single DENV strain per serotype/genotype was tested, which constitutes a bias of our experimental design considering the diversity of DENV serotype/genotype strains which circulate during an outbreak. Furthermore, the extrinsic incubation temperature may have an impact on the infection of the mosquito [33, 34] and on the transmission of arboviruses [35, 36]. Moreover, the infectious viral dose initially present in the blood meal may also affect the infection rate of *Ae*. *Aegypti* [37]. Yet another parameter to take into account is the natural multiple blood feeding behavior of *Ae*. *aegypti* which can increase the competence of this species for DENV [38, 39]. Furthermore, DENV co-infection experiments in *Ae*. *aegypti* can highlight significant competitive virus advantage, while no difference in vector competence is observed when mono-infection experiments are performed [40]. Finally, others factors such as virus genetics may have played an important role in the evolution of the dengue epidemiological profile in NC in the recent years [41]. Some studies have indicated that DENV genotype replacements were associated with differences in viral fitness, such as a higher viremia in humans [42] or a higher vectorial capacity [43–46]. In our study, no major difference was observed in the capacity of our local vector to transmit the four DENV serotypes. This indicates that factors related to humans would most probably best explain the persistence of DENV-1. All of these parameters should be taken into account in the future in order to better understand the relationship between the virus and the vector in a particular epidemiological context.

To conclude, our study provides the first experimental evidence of the ability of NC vector population *Ae*. *aegypti* to transmit all four DENV serotypes. Further studies on virus-vector interactions should be undertaken in the light of our results to better understand the DENV epidemiological profile in NC and the evolutionary dynamics of DENV at a larger scale. Indeed, DENV evolutionary dynamics are often characterized by DENV serotypic and/or genotypic replacements that can be epidemiologically significant. With only one proven vector for dengue in NC and the occurrence of DENV-1 genotype displacement in 2012, NC would be an ideal observatory to investigate the possible role of the vector in the selection of DENV variants and to better understand the evolutionary mechanisms driving DENV genotype replacement. Deepening our knowledge in this field is all the more important as DENV genotypes may differ in their antigenic properties and thus potentially have an impact on vaccine design.

## Supporting information

S1 Table Infection, dissemination, transmission efficiencies at 7 and 14 days post-infection (dpi) according to the four DENV serotypes. (DOCX)

S2 Table Log-linear model results according with the part of mosquitoes analyzed. (DOCX)

S3 Table Medians and interquartile ranges of viral titers measured per positive bodies, positive heads and positive saliva at 14 days post-infection (dpi) according to the four DENV serotypes. (DOCX)
