# Supplementary material for: Vector competence of Aedes aegypti from New Caledonia for the four recent circulating dengue virus serotypes
Source: PLoS Negl Trop Dis. 2020 May 14;14(5):e0008303. doi: 10.1371/journal.pntd.0008303 (PMC7252670; doi:10.1371/journal.pntd.0008303)
Supplement: S1 Table — (DOCX) [file pntd.0008303.s001.docx]

**Table S1: Infection, dissemination, transmission efficiencies at 7 and 14 days post-infection (dpi) according to the four DENV serotypes**

| **Viral strain** | **Days post-infection (dpi)** | **Number of individuals** | **% of infection** | **% of dissemination** | **% of transmission** | **% of efficiency** |
| --- | --- | --- | --- | --- | --- | --- |
| DENV-1 genotype I "Asia" | 7 | 45 | 22 (10/45) | 90 (9/10) | 22 (2/9) | 4 (2/45) |
| DENV-1 genotype IV "Pacific" | 7 | 48 | 40 (19/48) | 37 (7/19) | 14 (1/7) | 2 (1/48) |
| DENV-2 | 7 | 42 | 29 (12/42) | 58 (7/12) | 0 (0/7) | 0 (0/42) |
| DENV-3 | 7 | 38 | 18 (7/38) | 43 (3/7) | 0 (0/3) | 0 (0/38) |
| DENV-4 | 7 | 45 | 31 (14/45) | 43 (6/14) | 33 (2/6) | 4 (2/45) |
| DENV-1 genotype I "Asia" | 14 | 48 | 42 (20/48) | 100 (20/20) | 50 (10/20) | 21 (10/48) |
| DENV-1 genotype IV "Pacific" | 14 | 48 | 58 (28/48) | 93 (26/28) | 23 (6/26) | 13 (6/48) |
| DENV-2 | 14 | 51 | 43 (22/51) | 91 (20/22) | 40 (8/20) | 16 (8/51) |
| DENV-3 | 14 | 45 | 58 (26/45) | 49 (22/45) | 14 (3/22) | 7 (3/45) |
| DENV-4 | 14 | 45 | 20 (9/45) | 78 (7/9) | 43 (3/7) | 7 (3/45) |

Infection (number of infected bodies / number of individuals tested)

Dissemination (number of infected heads / number of infected bodies)

Transmission (number of infected saliva / number of infected heads)

Efficiency (number of infected saliva / number of individuals tested)
