# Supplementary material for: Vector competence of Aedes aegypti from New Caledonia for the four recent circulating dengue virus serotypes
Source: PLoS Negl Trop Dis. 2020 May 14;14(5):e0008303. doi: 10.1371/journal.pntd.0008303 (PMC7252670; doi:10.1371/journal.pntd.0008303)
Supplement: S2 Table — (DOCX) [file pntd.0008303.s002.docx]

**Table S2: Log-linear model results according with the different rates and efficiency analyzed (with 7 days post-infection as reference)**

| **Viral strains** | **Infection rate** | | | **Dissemination rate** | | | **Transmission rate** | | | **Transmission efficiency** | | |
| --- | --- | --- | --- | --- | --- | --- | --- | --- | --- | --- | --- | --- |
|  | **OR** | **95%CI** | ***p*-value** | **OR** | **95%CI** | ***p*-value** | **OR** | **95%CI** | ***p*-value** | **OR** | **95%CI** | ***p*-value** |
| DENV-1 genotype I "Asia" | 1.38 | [0.72-2.65] | 0.33 | **18.45** | **[1.97-172.81]** | **0.01** | **6.67** | **[1.57-28.42]** | **0.01** | **4.33** | **[1.15-16.2]** | **0.03** |
| DENV-1 genotype IV "Pacific" | **2.88** | **[1.53-5.40]** | **<0.001** | 1.27 | [0.39-4.13] | 0.7 | 2.17 | [0.5-9.54] | 0.3 | 2.28 | [0.56-9.27] | 0.25 |
| DENV-2 | 1.64 | [0.86-3.13] | 0.13 | 2.05 | [0.55-7.66] | 0.29 | 3.63 | [0.83-15.98] | 0.09 | 2.55 | [0.64-10.1] | 0.18 |
| DENV-3 | 1.90 | [0.99-3.65] | 0.05 | 1.01 | [0.26-3.88] | 0.99 | 1 | -- | -- | 1 | -- | -- |
| DENV-4 | 1 | -- | -- | 1 | -- | -- | **7.1** | **[1.27-39.79]** | **0.03** | 1.69 | [0.38-7.41] | 0.49 |

OR: Odds Ratio

Infection rate (number of infected bodies / number of individuals tested)

Dissemination rate (number of infected heads / number of infected bodies)

Transmission rate (number of infected saliva / number of infected heads)

Transmission efficiency (number of infected saliva / number of individuals tested)
