# Supplementary material for: Vector competence of Aedes aegypti from New Caledonia for the four recent circulating dengue virus serotypes
Source: PLoS Negl Trop Dis. 2020 May 14;14(5):e0008303. doi: 10.1371/journal.pntd.0008303 (PMC7252670; doi:10.1371/journal.pntd.0008303)
Supplement: S3 Table — (DOCX) [file pntd.0008303.s003.docx]

**Table S3: Medians and interquartile ranges of viral titers measured per positive bodies, positive heads and positive saliva at 14 days post-infection (dpi) according to the four DENV serotypes**

| **Viral strains** | **Day post-infection (dpi)** | **Positive Bodies** | | | **Positive Heads** | | | **Positive saliva** | | |
| --- | --- | --- | --- | --- | --- | --- | --- | --- | --- | --- |
|  |  | **Number** | **Median (FFU/mL)** | **Interquartile range (FFU/mL)** | **Number** | **Median (FFU/mL)** | **Interquartile range (FFU/mL)** | **Number** | **Median (FFU/mL)** | **Interquartile range (FFU/mL)** |
| DENV-1 genotype I "Asia" | 14 | 20 | 1900 | 10418 | 20 | 2150 | 2000 | 10 | 114 | 179 |
| DENV-1 genotype IV "Pacific" | 14 | 28 | 1250 | 1825 | 26 | 370 | 645 | 6 | 57 | 21 |
| DENV-2 | 14 | 22 | 122000 | 140500 | 20 | 17000 | 46700 | 8 | 128 | 243 |
| DENV-3 | 14 | 26 | 24000 | 68400 | 22 | 950 | 7105 | 3 | 86 | 143 |
| DENV-4 | 14 | 9 | 47000 | 99000 | 7 | 14000 | 34935 | 3 | 229 | 214 |

Medians and interquartile ranges are expressed in FFU/mL per bodies, heads or salivas according to the compartment of interest.
